# Epitaxial growth of ultrathin layers on the surface of sub-10 nm nanoparticles: the case of β-NaGdF_4_:Yb/Er@NaDyF_4_ nanoparticles

**DOI:** 10.1039/c8ra01752b

**Published:** 2018-04-06

**Authors:** Yang Su, Li-Na Hao, Kun Liu, Jun Zhang, Liang Dong, Yunjun Xu, Yang Lu, Hai-Sheng Qian

**Affiliations:** Department of Chemistry, Hefei University of Technology Hefei 230009 P. R. China yanglu@hfut.edu.cn; Department of Medical Materials and Rehabilitation Engineering, School of Medical Engineering, Hefei University of Technology Hefei 230009 P. R. China shqian@hfut.edu.cn +86 551 62901285; Analytical and Testing Center, Hefei University of Technology Hefei 230009 P. R. China; Hefei National Laboratory for Physical Sciences at the Microscale, University of Science and Technology of China Hefei 230026 P. R. China dldisc@ustc.edu.cn; Department of Radiology, Anhui Provincial Hospital Hefei 230001 P. R. China

## Abstract

Upconversion core–shell nanoparticles have attracted a large amount of attention due to their multifunctionality and specific applications. In this work, based on a NaGdF_4_ sub-10 nm ultrasmall nanocore, a series of core–shell upconversion nanoparticles with uniform size doped with Yb^3+^, Er^3+^ and NaDyF_4_ shells with different thicknesses were synthesized by a facile sequential growth process. NaDyF_4_ coated upconversion luminescent nanoparticles showed an obvious fluorescence quenching under excitation at 980 nm as a result of energy resonance transfer between Yb^3+^, Er^3+^ and Dy^3+^. NaGdF_4_:Yb,Er@NaDyF_4_ core–shell nanoparticles with ultrathin layer shells exhibited a better *T*_1_-weighted MR contrast.

## Abstract 2

In this work, a series of core–shell upconversion nanoparticles with uniform size doped with Yb^3+^, Er^3+^ and NaDyF_4_ shells with different thicknesses were synthesized by a facile sequential growth process.

## Graphical abstract

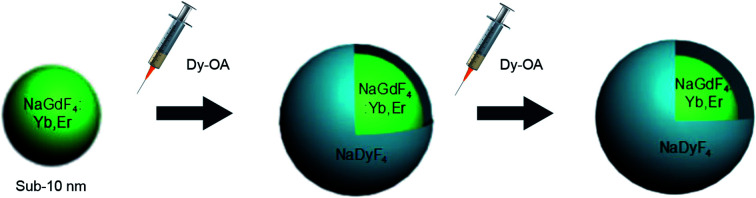

## Introduction

In recent years, core–shell nanoparticles with controllable sizes and shapes have attracted a great deal of interest because of their diverse applicability.^1–4^ Lanthanide-doped upconversion nanoparticles (UCNPs) have been studied widely due to their broad applications in energy and biological areas, such as biomedical imaging, solar energy conversion and photocatalysis.^5–13^ There are known methods for synthesizing core–shell nanoparticles with well-controlled sizes, shapes and nanostructures, which lead to enhanced performance.^14–17^ Most of these upconversion nanocrystals have been fabricated by a seed-mediated growth process, which is more complex and time-wasting. Moreover, various organic solvents were necessary. Recent reports indicate that the core–shell nanoparticles synthesized by the seed-mediated heat-up method were less likely to form a uniform shell, and the nanosized cores were only partially covered by a shell.^18,19^ Recently, with a high-boiling-point oleic complex serving as a shell precursor, a successive one-pot layer-by-layer (SLBL) technique has been developed to fabricate high-quality monodispersed core–shell NaGdF_4_:Yb,Er@NaYF_4_ upconversion nanoparticles.^20^ However, the synthesis of uniform ultrathin layers on the surface of sub-10 nm nanoparticles is still a challenge.

The fluorescence intensity of the core–shell structured upconversion nanoparticles was much stronger than the single core, because the shell can make up for the surface defect of the core.^21–23^ As an example, NaYF_4_:Yb,Tm@NaYF_4_:Yb,Er nanocrystals exhibited a remarkable enhancement in fluorescence intensity.^24^ With the introduction of an inert shell, ultrasmall LaF_3_:Yb^3+^/Ln^3+^ (Ln = Er, Tm, and Ho)@LaF_3_ core–shell nanoparticles exhibited a maximum 9 times enhancement of upconversion intensity compared with single nanocores.^16^ In general, lanthanide-doped upconversion nanoparticles NaGdF_4_:Yb,Er can emit visible light based on energy transfer under the excitation of a laser.^25^ As the main sensitizer, Yb^3+^ was excited by 980 nm photons and jumped from the ground state to the excited state (^2^F_5/2_), then jumped back to the ground state. The transition energy was then transferred to Er^3+^, which was excited from the ground level to an excited one. Subsequently, green emission at 522 nm and 547 nm ensues due to electrons in the excited state relaxing nonradiatively to lower-energy states (^2^H_11/2_ → ^4^I1_5/2_ and ^4^S_3/2_ → ^4^I1_5/2_).^26^ The core–shell structure of NaGdF_4_:Yb,Er@NaGdF_4_ nanoparticles showed a significant fluorescence enhancement.^27^ Nevertheless, NaDyF_4_ coated NaGdF_4_:Yb,Er nanoparticles presented an opposite result because Dy^3+^ is known as a quencher of the upconversion luminescence of Er^3+^.^28^ But there has been very little exploration into the Dy^3+^ quenching mechanism.

Magnetic resonance (MR) imaging is a useful invasive diagnostic technique used in clinical medicine. In order to enhance the contrast of the pathological region and diagnostic sensitivity, contrast agents (CAs) are frequently employed in MR diagnosis, and there are two kinds of MR contrast agents: positive (*T*_1_) CAs and negative (*T*_2_) CAs.^29–34^ The longitudinal relaxation time of the surrounding water protons could be effectively shortened by *T*_1_-weighted CAs (mostly paramagnetic Gd^3+^ chelates, such as Gd-DTPA), resulting in a brighter signal. The transverse relaxation time could be decreased by *T*_2_-weighted CAs (mostly superparamagnetic iron oxide (SPIO) nanoparticles), leading to a darker signal. In the clinic, most commercial contrast agents are gadolinium based contrast agents (GBCAs), because the brightened signals produced by GBCAs in the *T*_1_-weighted images of lesions are easily identified by clinicians. Due to the poor physiological stability of linear GBCAs,^35,36^ and their relative side-effects such as nephrogenic systemic fibrosis (NSF) in kidney patients,^37,38^ more efforts have been recently suggested for the development of GBCAs with a higher relaxivity and better thermodynamic stability. Very recently, ultrasmall sized NaGdF_4_ nanocrystals have exhibited great promise to serve as stable *T*_1_w MR contrast agents with a high *T*_1_w relaxivity and negligible leakage of free gadolinium ions.^39–42^ When the ultrasmall sized NaGdF4 nanocrystals serve as a core to fabricate core–shell structured nanocomposites, it’s reasonable that the *T*_1_w MR contrast performance should be affected due to the change in surface properties and less interaction with the surrounding protons. However, this remains unexplored.

Herein, a facile sequential growth process has been used to synthesize NaGdF_4_:Yb,Er@NaDyF_4_ core–shell nanoparticles with different shell thicknesses. NaGdF_4_:Yb,Er@NaDyF_4_-1 with ultrathin shells exhibited a higher longitudinal relaxivity (*r*_1_) than NaGdF_4_:Yb,Er@NaDyF_4_-2, which is a promising *T*_1_ magnetic resonance imaging agent. In addition, the cause of the decrease in upconversion fluorescence intensity of the NaDyF_4_ coated NaGdF_4_:Yb,Er was illustrated, which provides guidance for subsequent research into upconversion luminescent materials with core–shell structures.

## Experimental section

### Reagents and materials

DyCl_3_·6H_2_O(99.99), GdCl_3_·6H_2_O (99.99%), YbCl_3_·6H_2_O (99.99%) and ErCl_3_·6H_2_O (99.99%), sodium hydroxide (NaOH), ammonium fluoride (NH_4_F), oleic acid (OA, 90%) and 1-octadecene (ODE, 90%) were purchased from Aladdin Chemical Reagent Corporation (Shanghai, China). Organic reagents including methanol, cyclohexane and ethanol were purchased from Sinopharm Chemical Reagent Corporation (Shanghai, China). HepG2 cells were purchased from the Shanghai Institute of Cell Bank.

### Synthesis of Gd/Yb/Er-oleic and Dy-oleic complexes

195 mmol GdCl_3_, 0.05 mmol YbCl_3_, 0.005 mmol ErCl_3_ and 3 mL of OA were added into a 50 mL three-neck flask then heated to 100 °C and kept at this temperature for 20 minutes to remove the water during crystallization. The mixture was continuously stirred and heated for 20 minutes to form a clear solution (Gd/Yb/Er-OA complex). The Dy-oleic complex was prepared by a similar process using DyCl_3_ (0.125 mmol) and OA (3 mL). The Gd/Yb/Er-oleic and Dy-oleic complex solutions were stable, and were used as precursors of further reactions.

### Synthesis of NaGdF_4_:Yb,Er@NaDyF_4_ core–shell nanoparticles with various shell thicknesses

In a typical experiment, NaGdF_4_:Yb,Er@NaDyF_4_ core–shell nanoparticles were synthesized on the basis of a reported method with some changes.^43^ 6 mL OA and 15 mL ODE were added into a three-neck reaction flask, and a clear methanol solution (10 mL) containing 1.5 mmol of NaOH and 2 mmol of NH_4_F were added into the flask with vigorous stirring. Then the solution was slowly heated to 100 °C to remove methanol and water. After the water and oxygen was removed under vacuum with continuous stirring, the solution was heated to 280 °C slowly under an argon atmosphere. Then the Gd/Yb/Er-OA complex (0.25 mmol) precursor solution was injected into the reaction system quickly at 280 °C and maintained at this temperature for 60 min. The reaction solution was allowed to cool to room temperature then 0.125 mmol of the Dy-OA complex was injected rapidly into the reaction mixture at room temperature and the solution was heated to 280 °C for another 60 min to obtain the NaGdF_4_:Yb,Er@NaDyF_4_-1 with ultrathin shells. Finally, the solution was cooled down to room temperature naturally and the resulting nanoparticles were precipitated by the addition of ethanol, collected by centrifugation and then redispersed in cyclohexane.

NaGdF_4_:Yb,Er@NaDyF_4_-2 nanoparticles with different shell thicknesses were synthesized using the same process as for the abovementioned nanoparticles, except for one thing: after the first Dy-OA (0.125 mmol) compound was injected and incubated for 60 minutes, the reaction system was reduced to room temperature and 0.125 mmol of the Dy-OA complex was injected again, and kept at 280 °C for an additional 1 h.

### Surface modification

The hydrophobic NaGdF_4_:Yb,Er@NaDyF_4_ nanoparticles covered with oleic acid were transferred to the aqueous phase using a ligand-exchange method.^23^ NaGdF_4_:Yb,Er@NaDyF_4_ cyclohexane solution (10 mg mL^−1^ 10 mL) was dropwise added to 10 mL of PEG-PAA in 1,4-dioxane (10 mg mL^−1^) and stirred for 12 hours at room temperature. Then the mixed solution was naturally cooled down to room temperature after removing the cyclohexane at 80 °C. The resulting solution was collected by centrifugation and washed with water three times. The final hydrophilic nanoparticles were dispersed in water for further use.

### MR measurement

Various concentrations of the PEG–PAA modified NaGdF_4_:Yb,Er@NaDyF_4_ and NaGdF_4_:Yb,Er@NaDyF_4_@NaDyF_4_ nanoparticles dispersed in deionized water were prepared, and their *T*_1_ weighted MR images were acquired on a Siemens 3.0 Tesla MR scanner using the inversion recovery (IR) sequence. The imaging parameters were as follows: repetition time (*T*_R_) = 4000 ms, echo time (*T*_E_) = 10.88 ms, and a series of inversion times (*T*_I_) between 50 and 3500 ms were employed. The field of view (FOV) = 220 × 220 mm^2^. Then the *T*_1_ values of each sample at different concentrations were calculated on a workstation to obtain the relaxivity.

### Characterization

Transmission electron microscopy (TEM) analysis was carried out on a JEM-2100F (JEOL, Japan) transmission electron microscope. The phase of the as-prepared product was characterized by X-ray powder diffraction (XRD) analysis, which was performed on a Philips X’Pert PRO SUPER X-ray diffractometer equipped with graphite monochromated Cu Kα radiation, and the operation voltage and current were maintained at 40 kV and 40 mA, respectively. The upconversion fluorescence spectra were measured on an F-2700 fluorescence spectrometer (Hitachi High-Technology Corporation), where an external CW laser at 980 nm replaced the xenon lamp as the excitation source. Inductively coupled plasma atomic emission spectroscopy (ICP-AES) was employed to determine the concentration of Gd^3+^ in the solutions. FT-IR spectra were measured on a Bruker Vector-22 FT-IR spectrometer. The relaxivity measurements were carried out on a clinical MRI instrument (Siemens Trio Tim 3.0 T, Germany).

## Results and discussion

### Fabrication and epitaxial growth of β-NaGdF4:Yb/Er@NaDyF4 nanoparticles with an ultrathin layer

**Fig. 1:**
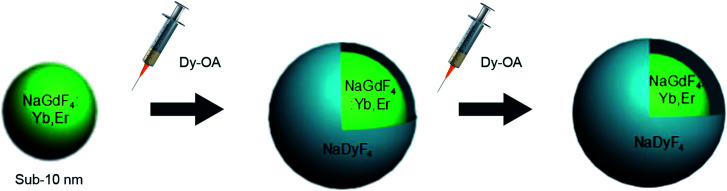
A schematic illustration of the epitaxial growth of NaGdF_4_:Yb/Er@NaDyF_4_ nanoparticles with different shell thicknesses.

The synthesis of NaGdF_4_:Yb/Er@NaDyF_4_ core–shell nanoparticles with different shell thicknesses was achieved by a sequential growth process (Fig. 1). NaGdF_4_:Yb,Er nanoparticles were first synthesized at 280 °C in the presence of sodium hydroxide, ammonium fluoride and Gd/Yb/Er-OA complexes.

When the reaction system containing NaGdF_4_:Yb,Er nanoparticles was reduced to room temperature, the Dy-OA (0.125 mmol) complex was injected, the solution was then heated to 280 °C and kept for one hour to obtain NaGdF_4_:Yb/Er@NaDyF_4_-1. Afterwards, another 0.125 mmol Dy-OA complex was injected quickly to the reaction system to synthesize the NaGdF_4_:Yb,Er@NaDyF_4_-2. Finally, NaGdF_4_:Yb,Er@NaDyF_4_ core–shell nanoparticles with different shell thicknesses could be obtained.

**Fig. 2:**
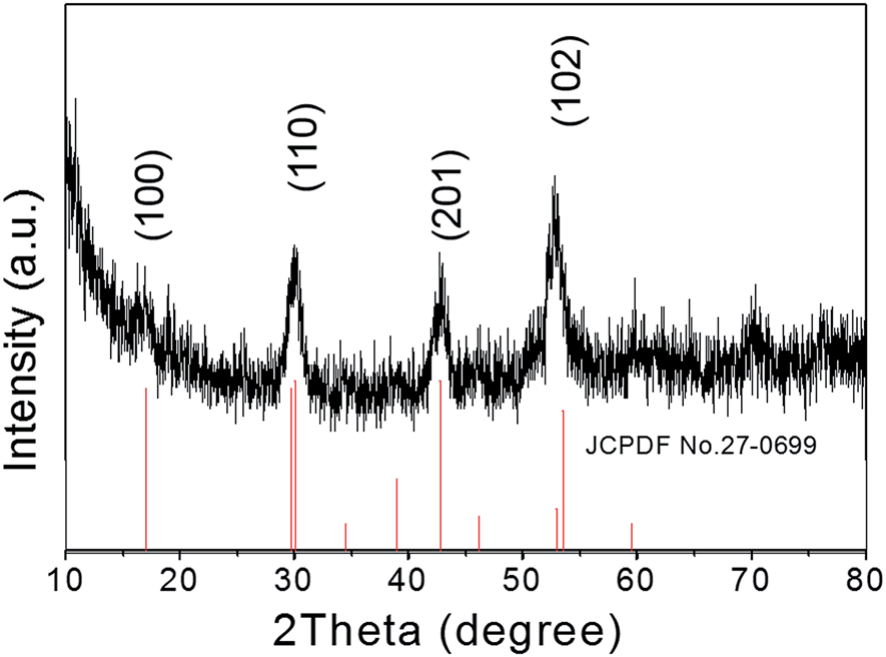
X-ray diffraction patterns of NaGdF_4_:Yb,Er@NaDyF_4_ nanoparticles.

The crystallographic structures of the as-prepared NaGdF_4_:Yb,Er@NaDyF_4_ nanoparticles were studied by X-ray powder diffraction (XRD) (Fig. 2). All of the identified diffraction peaks for the core–shell nanoparticles in the XRD pattern were in accordance with the data for the reference hexagonal phase of NaGdF4 (JCPDS card no. 27-0699). No impurity crystalline phase was found in the diffraction pattern. The broadening of the diffraction peaks ascribed to OA-coated nanoparticles distinctly indicated the property of nanocrystallinity. Therefore, the XRD pattern revealed that the composition of OA-coated nanoparticles is very pure.

**Fig. 3:**
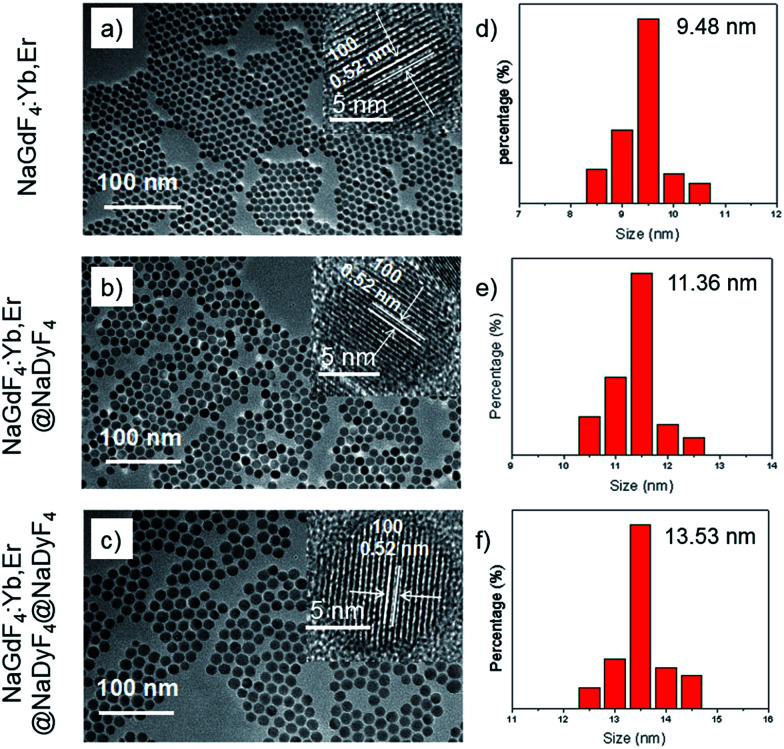
TEM images and the size distribution of (a and d) NaGdF_4_:Yb,Er, (b and e) NaGdF_4_:Yb,Er@NaDyF_4_-1 and (c and f) NaGdF_4_:Yb,Er@NaDyF_4_-2. The insets of (a–c) are HR-TEM images of the above three nanoparticles and the scale bars correspond to 5 nm.

Transmission electron microscopy (TEM) was used to study the structures and morphology of the as-synthesized nanoparticles (Fig. 3a–c). The NaGdF_4_:Yb,Er nanoparticles with a uniform size of about 9.5 nm (Fig. 3a and d) are monodispersed well. After the NaGdF_4_:Yb,Er cores were coated by NaDyF_4_ shells, the diameters of the nanoparticles were distributed mainly at about 11.5 nm. The thickness of the NaDyF4 shell was about 1 nm (Fig. 3b and e). After further growth of NaDyF_4_ shells on the surface of NaGdF_4_:Yb,Er@NaDyF_4_-1, the monodisperse nanoparticles exhibited an average size of 13.5 nm, indicating that NaDyF_4_ nanocrystals with about 2 nm shell thickness were grown on the surface of NaGdF_4_:Yb,Er nanocores (Fig. 3c and f).

**Fig. 4:**
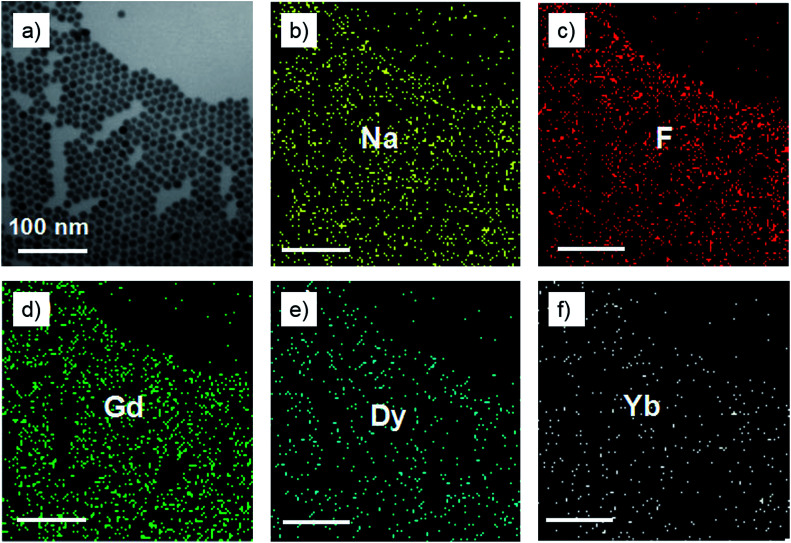
(a) A scanning transmission electron microscopy (STEM) image of the as-prepared core–shell nanoparticles of NaGdF_4_:Yb,Er@NaDyF_4_. (b–f) Elemental mapping images of Na, F, Gd, Dy and Yb for the STEM image, respectively.

**Fig. 5:**
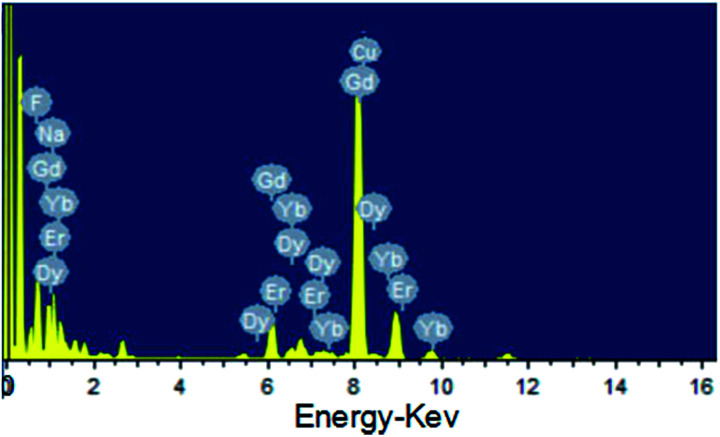
The energy dispersive X-ray analysis (EDX) of the as-prepared NaGdF_4_:Yb,Er@NaDyF_4_ nanoparticles.

**Fig. 6:**
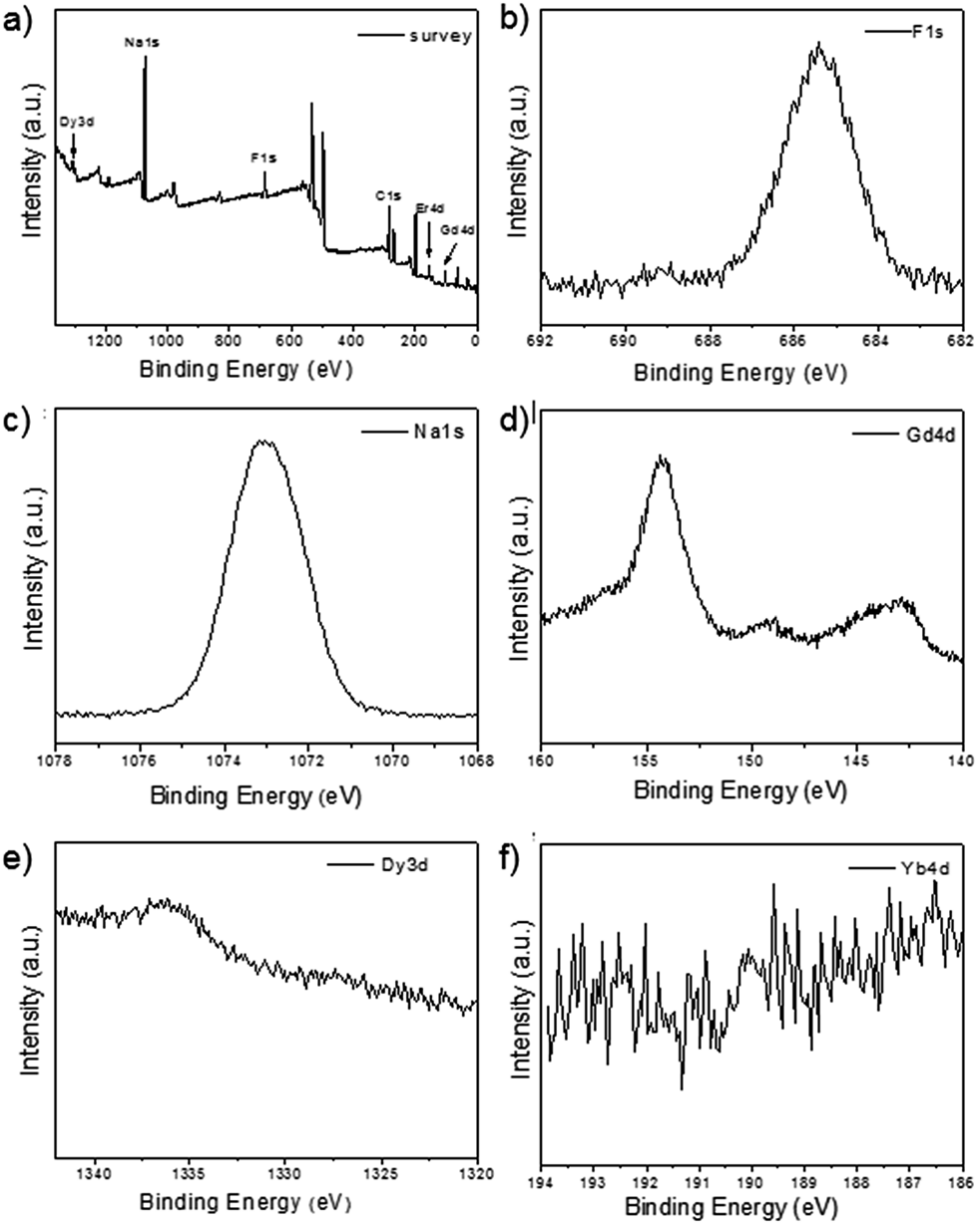
The X-ray photoelectron spectroscopy (XPS) analysis of NaYF_4_:Yb,Er@NaGdF_4_. (a) The survey spectrum; (b) F 1s; (c) Na 1s; (d) Gd 4d; (e) Dy 3d; (f) Yb 4d.

As shown in the insets of Fig. 3a–c, the high-resolution transmission electron microscopy (HR-TEM) images taken of the enlarged single nanoparticles of the above three samples proved that the nanoparticles had high crystallinity with a lattice spacing of 0.52 nm, which was consistent with the (100) plane of the NaGdF_4_ nanoparticles. Fig. 4a shows a scanning transmission electron microscopy image of the as-prepared NaGdF_4_:Yb,Er@NaDyF_4_ nanocrystals. The elemental mapping images for Na, F, Gd, Dy and Yb are shown in Fig. 4b–f, indicating the co-existence of these elements in the as-prepared nanoparticles of NaGdF_4_:Yb,Er@NaDyF_4_. As shown in Fig. 5, energy dispersive X-ray analysis proved the co-existence of Na, F, Gd, Dy and Yb in the as-synthesized nanoparticles, and the calculated chemical composition has been shown in Table 1. The survey scan X-ray photoelectron spectroscopy (XPS) spectrum, as shown in Fig. 6, of the core–shell nanoparticles showed photoelectron lines at binding energies of about 100 eV, 710 eV, 1080 eV, and 1300 eV, attributed to Gd 4d, F 1s, Na 1s, and Dy 3d, respectively. This is in good agreement with the results of the elemental mapping and EDX. All of these results indicated that the core–shell nanoparticles of NaGdF_4_:Yb,Er @NaDyF_4_ have been synthesized successfully.

**Table 1:** The element composition of the as-prepared NaGdF_4_:Yb,Er@NaDyF_4_ core–shell nanoparticles from EDX analyses

Element	Weight%	Atomic%
F	23.62	60.45
Na	9.06	19.17
Gd	42.15	13.03
Dy	15.22	4.55
Er	0.00	0.00
Yb	9.94	2.79
Totals	100	

### UC fluorescence of β-NaGdF4:Yb/Er@NaDyF4 nanoparticles with an ultrathin layer

**Fig. 7:**
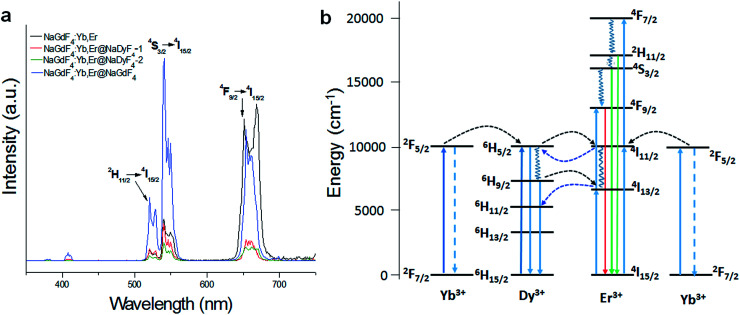
(a) The upconversion emission spectra of synthesized NaGdF_4_:Yb,Er, NaGdF_4_:Yb,Er@NaDyF_4_-1 and NaGdF_4_:Yb,Er@NaDyF_4_-2 nanoparticles under the excitation of 980 nm. (b) The proposed energy transfer mechanism in NaDyF_4_ coated nanoparticles.

Fig. 7a shows the UC emission spectra of the different nanoparticles excited at 980 nm. The UC fluorescence peaks are located at 520, 540 and 656 nm, and were assigned to the transitions from ^2^H_11/2_, ^4^S_3/2_, and ^4^F_9/2_ to ^4^I_15/2_ of Er, respectively.^44^ The presence of Dy^3+^ in the shell has shown an obvious influence on the UC luminescence intensity, with the luminescence intensity of NaGdF_4_:Yb,Er-1 and NaGdF_4_:Yb,Er@NaDyF_4_-2 being much lower than that of NaGdF_4_:Yb,Er. In contrast, the luminescence intensity of NaGdF_4_:Yb,Er@NaGdF_4_ was investigated as well, and was much stronger than that of NaGdF_4_:Yb,Er@NaDyF_4_. The result indicated that the presence of Dy^3+^ in the shell induced a noticeable quenching of Er^3+^ luminescence.

One explanation for the Dy^3+^ quenching of Er^3+^ luminescence is the depopulation of ^4^I_11/2_ (Er^3+^) and ^2^F_5/2_ (Yb^3+^) by Dy^3+^.^45^ The energy transfer between Dy^3+^, Yb^3+^, and Er^3+^ can readily occur as the result of the ^6^H_5/2_ → ^6^H_15/2_ transition of Dy^3+^, which is resonant with the ^2^F_5/2_ → ^2^F_7/2_ transition of Yb^3+^ ions and the ^4^I_11/2_ → ^4^I_15/2_ transition of Er^3+^ ions (Fig. 7b). Dy^3+^ can be excited by 980 nm photons from the ^6^H_15/2_ ground state to the ^6^H_5/2_ excited state or receive the energy from excited Yb^3+^ and Er^3+^ ions. The life time of ^6^H_5/2_ is short, and the back-energy transfer to Yb^3+^ is so little that it can be neglected.^46,47^ The excited Dy^3+^ can relax radiatively to the ground state, and divert some energy to Er^3+^ to cause Er^3+^ excitation from the ground state (^4^I_15/2_) to the excited state and then to the upper excitation level. Subsequently, a red emission around 660 nm takes place caused by a radiative transition from the ^4^F_9/2_ to ^4^I_15/2_ level. Resonance energy transfer has been demonstrated to study the Dy^3+^ quenching of Er^3+^ luminescence. Yb^3+^, as a sensitizer, is excited at 980nm from the ground state ^2^F_7/2_ to the only excitation level ^2^F_5/2_ then transmits energy to the activator Er^3+^. Yb^3+^ has a much larger absorption cross-section than Dy^3+^, so Yb^3+^ can offer more energy to Er^3+^ at this level. Since NaGdF_4_:Yb,Er@NaDyF_4_@NaDyF_4_ has a thicker NaDyF_4_ layer on the surface of NaGdF_4_:Yb,Er, the fluorescence intensity is quite weaker.

### The MR contrast performance of β-NaGdF4:Yb/Er@NaDyF4 nanoparticles with an ultrathin layer

**Fig. 8:**
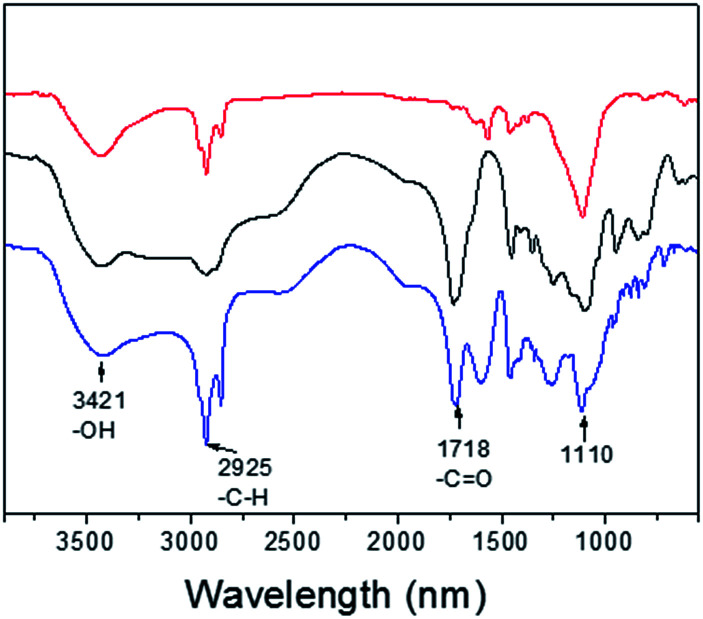
The responsive Fourier transform infrared spectra (FTIR) of UCNPs-OA (red line), PEG-PAA (black line) and PEG-PAA coated UCNPs (blue line).

To investigate the MRI performance of the nanoparticles with different shell thicknesses, we conducted relaxivity measurements on a 3.0 T MRI clinical scanner. We measured the gadolinium concentration of PEG-PAA modified NaGdF_4_:Yb,Er@NaDyF_4_ and NaGdF_4_:Yb,Er@NaDyF_4_@NaDyF_4_ by inductively coupled plasma-atomic emission spectrometry (ICP-AES). FT-IR has been performed to prove that hydrophobic upconversion core–shell nanoparticles were successfully modified by PEG-PAA in a ligand exchange way. As shown in Fig. 8, two stronger peaks at 1555 and 1456 cm^−1^ could be observed, corresponding to the asymmetric and symmetric stretching vibration of the COO^−^ group, respectively. After being modified with PEG-PAA, there are two new bands at 1718 and 1110 cm^−1^, which could be attributed to the stretching vibration of C=O and C–O of PEG-PAA chains, respectively.

**Fig. 9:**
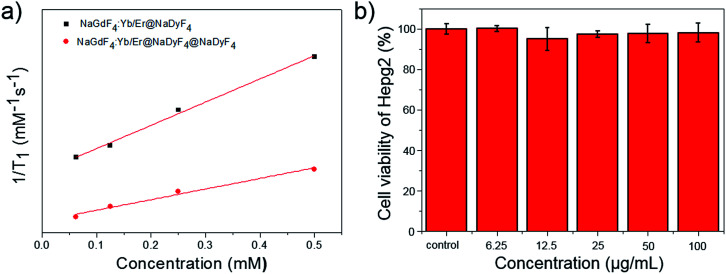
(a) A comparison of the longitudinal relaxivity of PEG-PAA modified NaGdF_4_:Yb,Er@NaDyF_4_ and NaGdF_4_:Yb,Er@NaDyF_4_@NaDyF_4_. (b) The cyto-compatibility result of PEG-PAA modified NaGdF_4_:Yb,Er@NaDyF_4_ on HepG2 cells.

As is well known, paramagnetic Gd^3+^ ion based materials including Gd^3+^ ions and chelating ligands are *T*_1_ (positive) contrast agents. The longitudinal relaxation times (*T*_1_) of different concentrations of PEG-PAA modified nanoparticles in solution were examined by a 3.0 T clinical MR scanner using a standard inversion recovery sequence. As shown in Fig. 9a, the *T*_1_ value was significantly reduced with increasing Gd^3+^ concentration, indicating an obvious enhancement of *T*_1_ weighted MR contrast. The *r*_1_ of NaGdF_4_:Yb,Er@NaDyF_4_ nanoparticles with a thin layer is 1.36 mM^−1^ s^−1^, which is 2-fold higher than that of NaGdF_4_:Yb,Er@NaDyF_4_@NaDyF_4_ with a thicker shell (0.62 mM^−1^ s^−1^), indicating that NaGdF_4_:Yb,Er@NaDyF_4_ is a better *T*_1_ contrast agent than NaGdF_4_:Yb,Er@NaDyF_4_@NaDyF_4_. In addition, the cytotoxicity of the NaGdF_4_:Yb,Er@NaDyF_4_ nanoparticle was evaluated by a standard MTT method. As shown in Fig. 9b, the viability of HepG2 cells did not exhibit any reduction after exposure to NaGdF_4_:Yb,Er@NaDyF_4_ nanoparticles for 24 hours, which confirmed their promising cyto-compatibility.

## Conclusions

In conclusion, NaGdF_4_:Yb,Er coated with different thickness layers of NaDyF_4_ core–shell nanoparticles have been synthesized successfully by a facile sequential growth process with the low temperature injection of a Dy-OA complex, which had a uniform size and shape. The excitation of the synthesized nanoparticles at 980 nm showed an obvious luminescence quenching arising out of energy migration-mediated upconversion. Furthermore, ultrathin NaDyF_4_ shell coating remained the *T*_1_-weighted MR imaging performance of NaGdF_4_:Yb,Er.

## Conflicts of interest

There are no conflicts to declare.

## Supplementary Material
